# Genomic instability and cellular stress in organ biopsies and peripheral blood lymphocytes from patients with colorectal cancer and predisposing pathologies

**DOI:** 10.18632/oncotarget.4032

**Published:** 2015-05-23

**Authors:** Sara Lombardi, Ilenia Fuoco, Giorgia di Fluri, Francesco Costa, Angelo Ricchiuti, Graziano Biondi, Vincenzo Nardini, Roberto Scarpato

**Affiliations:** ^1^ Unità di Genetica, Dipartimento di Biologia, University of Pisa, Pisa, Italy; ^2^ Azienda Ospedaliera Universitaria Pisana, Unità Operativa di Gastroenterologia, Pisa, Italy; ^3^ Azienda USL 5, Unità Operativa Chirurgia, Ospedale F. Lotti, Pontedera, Italy; ^4^ Azienda Ospedaliera Universitaria Pisana, Unità Operativa di Anatomia e Istologia Patologica 2, Pisa, Italy; ^5^ Research Center Nutraceuticals and Food for Health-Nutrafood, University of Pisa, Pisa, Italy

**Keywords:** colorectal cancer, genomic instability, γH2AX, GSTO1, micronuclei

## Abstract

Inflammatory bowel disease (IBD) and polyps, are common colorectal pathologies in western society and are risk factors for development of colorectal cancer (CRC). Genomic instability is a cancer hallmark and is connected to changes in chromosomal structure, often caused by double strand break formation (DSB), and aneuploidy. Cellular stress, may contribute to genomic instability. In colorectal biopsies and peripheral blood lymphocytes of patients with IBD, polyps and CRC, we evaluated 1) genomic instability using the γH2AX assay as marker of DSB and micronuclei in mononuclear lymphocytes kept under cytodieresis inhibition, and 2) cellular stress through expression and cellular localization of glutathione-S-transferase omega 1 (GSTO1). Colon biopsies showed γH2AX increase starting from polyps, while lymphocytes already from IBD. Micronuclei frequency began to rise in lymphocytes of subjects with polyps, suggesting a systemic genomic instability condition. Colorectal tissues lost GSTO1 expression but increased nuclear localization with pathology progression. Lymphocytes did not change GSTO1 expression and localization until CRC formation, where enzyme expression was increased. We propose that the growing genomic instability found in our patients is connected with the alteration of cellular environment. Evaluation of genomic damage and cellular stress in colorectal pathologies may facilitate prevention and management of CRC.

## INTRODUCTION

Colorectal cancer (CRC) is one of the most frequent cancer type in western society. Heredity, age, sex, race, lifestyle and the presence of inflammatory bowel diseases (IBD) are considered to increase the risk of CRC [1]. For decades, the adenoma-carcinoma sequence has been taken as a paradigm for studies on CRC progression [2]. In the sequence, the formation of polyps into colorectal mucosa represents the pre-cancerous state, an event associated to mutation in crucial genes such as APC. Among the different histological types that have been described, adenomatous polyps are considered the most frequent and dangerous. Recently, the classical adenoma-carcinoma sequence has been reviewed, including IBD as CRC predisposing pathologies. The most frequently observed IBD are ulcerative colitis (UC) and Crohn's disease (CD), both characterized by intermittent inflammatory states that influence the risk of cancer progression, especially in relation to the duration of the inflammatory state [3]. IBD are probably connected to CRC via genomic instability, a hallmark of cancer phenotype that manifests in several different ways, and oxidative stress. Both conditions are strictly related to each other and often have a synergistic effect.

Interruption of the sugar-phosphate skeleton in both strands is considered the most dangerous DNA lesion. When a double strand break (DSB) occurs, in fact, a complex signaling pathway, the DNA damage response (DDR), is activated promoting repair of the lesion, when possible, or addressing cells to apoptosis, if the damage is too extended. One of the first events of the DDR is the phosphorylation at Ser 139 of H2AX histone variant mainly by the ataxia telengiectasia mutated (ATM) kinase to generate the γH2AX form [4]. There is a positive feedback among the DDR initiator factors that promotes the autoamplification of γH2AX. The great amount of γH2AX, which forms immediately after the DSB formation, develops in a γH2AX focus, which covers a chromatin region of several Mbp [5]. As γH2AX foci disappear immediately after DSB is repaired, they can efficiently be used as a quantitative marker of DSB. The γH2AX focus assay is therefore being used in a multitude of genotoxicity studies as well as in clinical investigations to assess pathology effect on genomic integrity [6], [7], [8]. Understanding in which pathological stages the first steps of DDR are impaired may be useful in diagnostic and in therapy modulation, as this response is considered an anti-cancer barrier that is gradually lost during the progression of most cancers [9]. Genomic instability is also connected to a higher rate of chromosome number alterations at each cell division, caused by bad functioning in the spindle apparatus, thus leading to aneuploidy. A universally accepted marker of genomic instability is the frequency of micronuclei (MN) that form within proliferating cells after chromosome breakage or chromosome malsegregation. The Cytokinesis Block Micronucleus assay (CBMN) is a standardized test in which MN frequency is evaluated in the population of binucleated cells obtained by the use of cytochalasin B (cyt B) as cytodieresis inhibitor [10]. However, the exposure of cells to aneugens like spindle poisons is known to induce, despite the presence of cyt B, a high proportion of mononucleated cells with MN [11] which are able to survive and generate aneuploid cells, in subsequent divisions. Thus, these cells can be used as a marker of predisposition to aneuploidy onset [12], [13].

Oxidative stress is a hallmark of several pathologies and, due to the mutagenic capacity of ROS, it has also been associated with cancer progression [14]. The glutathione-S-transferase omega class (GSTO), a member of glutathione-S-transferases family participating in detoxification, anti-oxidative defense as well as in important cellular signaling pathways, comprises GSTO1 and GSTO2 isoforms [15], [16]. In particular, as GSTO1 is active in dehydroascorbate reduction and in the regulation of several targets due to its de-glutathionylation capacity, it is considered to be involved in the anti-oxidative defense and in cellular signaling more than in xenobiotics metabolism [15], [17]. In addition, GSTO1 nuclear relocation has been reported in response to heat shock as well as in Barrett's esophagus [18], [19].

Keeping all this in mind, the aim of this work is evaluating DNA damage, predisposition to aneuploidy and cellular stress response in pathological progression to CRC through the study of γH2AX, MN frequency in mononucleated cells and GSTO1 behavior (expression and relocation). This was performed in groups of subjects representative of the pathological sequence leading to CRC, including IBD and polyps as intermediate states. The above mentioned markers were analyzed in colorectal epithelium, directly involved in the pathologies, and in peripheral blood lymphocytes, a healthy cellular system that may reflect the events occurring in the intestinal mucosa, due to their circulating and surveillance activity.

## RESULTS

### Colorectal biopsies results

#### γH2AX

**Figure 1:**
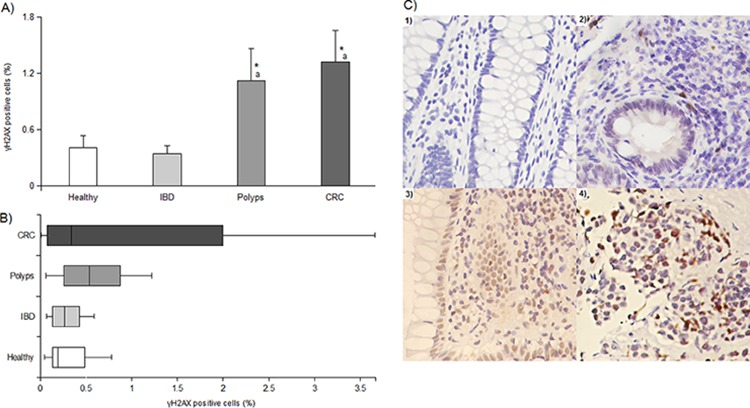
γH2AX immunohistochemistry results **Panel A.** average levels of γH2AX in the study population expressed as percentage of positive cells; * and *a* indicate a significant difference (*P* < 0.01) from healthy tissue and IBD samples, respectively. In each group, bars represent the mean ± SE. **Panel B.** box and whisker plot displaying data dispersion for γH2AX in each category. **Panel C.** photos of γH2AX expression in tissues from 1) healthy, 2) IBD, 3) polyp and 4) cancer biopsies. 200x magnification.

The results of multivariate analysis in tissue samples, corrected for sex and age, is visualized in Figure 1A. Polyps (1.12 ± 0.34%) and CRC (1.32 ± 0.34%) show significantly (*P* < 0.01) higher phosphorylation levels than healthy (0.41 ± 0.12%) and IBD (0.34 ± 0.08%) samples. A deeper observation of data distribution in the study population, as graphically visualized in the box and whisker plots (Figure 1B), reveals a strong dispersion of the single observations for CRC and polyp categories with respect to IBD and healthy biopsies. In addition, the γH2AX percentage of positive cells that limits the lower quartile of CRC is lower (0.08%) than the one observed in healthy tissue (0.13%) while, in polyps, this value is the highest (0.26%). Anatomical biopsy provenience does not have an effect on γH2AX even if, proceeding to more distal intestinal regions, we observed a slight increase in H2AX phosphorylation, suggesting a higher sensitivity of these districts (data not shown). We did not find significant differences either between dysplasic and hyperplastic polyps or within IBD pathologies although H2AX phosphorylation levels doubled in CD (0.49 ± 1.17%) with respect to UC (0.24 ± 0.05%) (data not shown). Qualitatively, the superficial epithelium is the colorectal mucosa portion principally involved in γH2AX formation, with some positive nuclei on the base of the crypt, near the stem cell compartment. In the IBD sections we observed a principal positivity of the stromal portion of mucosa, probably in connection with the immunity cellular infiltration in this region (Figure 1C).

#### GSTO1

**Figure 2:**
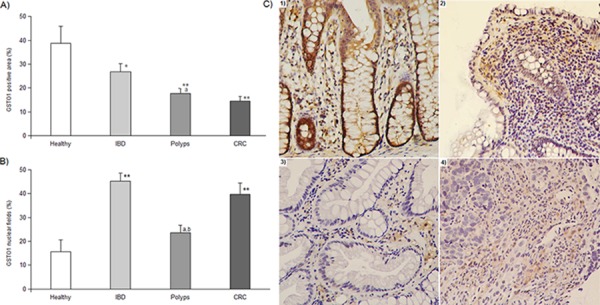
GSTO1 immunohistochemistry results **Panel A.** GSTO1 expression expressed as percentage of positive area. In each group, bars represent the mean ± SE. **Panel B.** GSTO1 localization results expressed as percentage of nuclear fields. * and ** indicate a significant difference from healthy samples with *P* < 0.01 and *P* < 0.001, respectively; *a* and *b* indicate a significant difference (*P* < 0.01) from IBD and CRC category, respectively. In each group, bars represent the mean ± SE. **Panel C.** photos of GSTO1 expression in tissues from 1) healthy, 2) IBD, 3) polyp and 4) cancer biopsies. 100x magnification.

Compared to healthy tissues that display the highest positivity level (38.79 ± 7.14%), GSTO1 expression gradually and significantly decreases in IBD (26.79 ± 3.42%, *P* < 0.01), in adenomas (17.70 ± 2.19%, *P* < 0.001) and in cancer (14.55 ± 1.87%, *P* < 0.001) (Figure 2A). In addition, statistical analysis shows that adenomas are distinct from IBD (*P* < 0.01), but not from carcinomas. The highest levels of nuclear GSTO1 localization is obtained in IBD (45.28 ± 3.42%) and CRC (39.78 ± 4.75%) samples that are significantly higher than those found in healthy biopsies (15.62 ± 5.07%, *P* < 0.001) and polyps (23.62 ± 3.11%, *P* < 0.01) (Figure 2B). No effect of sex, age, anatomical district provenience, histological polyp type or inflammatory pathology on expression and relocation of GSTO1 were observed, even though more distal intestine portions show a more pronounced decrease in expression and increase in nuclear localization. Qualitatively, we observed that GSTO1 is generally expressed in the apical stroma, and only in IBD also an epithelial expression was found (Figure 2C).

### Peripheral lymphocytes results

#### γH2AX

**Figure 3:**
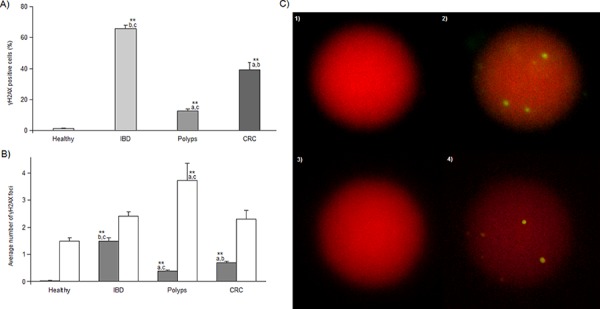
γH2AX immnofluorescence results **Panel A.** γH2AX percentage of positive cells in each group expressed as mean ± SE. **Panel B.** average number of γH2AX foci per cell (gray bars) or per positive cell (white bars) in each group expressed as mean ± SE. ** indicate a significant difference (*P* < 0.001) from healthy subjects; *a*, *b* and *c* indicate a significant difference (*P* < 0.001) from IBD, polyps and CRC category, respectively. **Panel C.** photos of two γH2AX positive nuclei counterstained with propidium iodide and visualized using filters for TRITC (1, 3) or FITC (2, 4) where three (2) and six (4) γH2AX foci are present (green-yellow spots). 1000x magnification.

Statistical analysis shows that all the mean percentage of γH2AX positive cells found in the pathological groups are significantly different (*P* < 0.001) from the one of healthy subjects (1.25 ± 0.38%) and among them. In particular, γH2AX positive cells reach the highest value (65.90 ± 2.15%, *P* < 0.001) in IBD subjects, while they gradually and significantly (*P* < 0.001) rise in polyps (12.59 ± 1.21%) and CRC affected patients (39.10 ± 4.72%) (Figure 3A). A similar trend is delineated for the levels of γH2AX foci per cell (healthy subjects = 0.03 ± 0.01, polyps = 0.38 ± 0.06, CRC = 0.69 ± 0.06; IBD =1.49 ± 0.12, *P* < 0.001) (Figure 3B). At variance, the average number of γH2AX foci per positive cell is 1.49 ± 0.11 in healthy subjects, 2.40 ± 0.17 in IBD and it significantly increases in the polyp group (3.73 ± 0.63, *P* < 0.001), while in CRC (2.29 ± 0.32) it falls down to the levels observed in healthy and IBD categories (Figure 3B). Neither sex nor age affected all the three γH2AX parameters considered.

#### GSTO1

**Figure 4:**
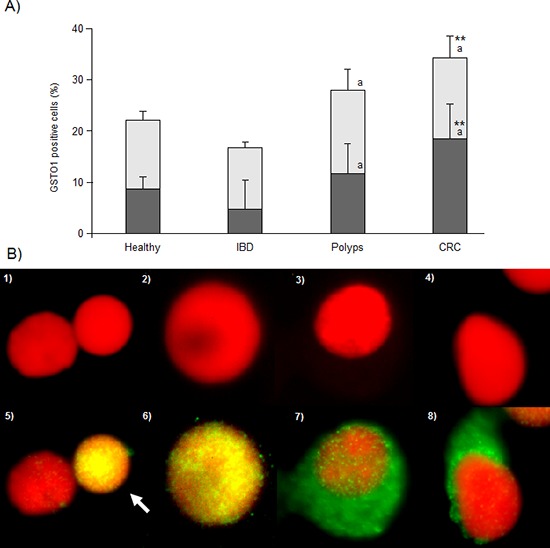
GSTO1 immunofluorescence results **Panel A.** percentage of cells positive for GSTO1 (light bars) or showing nuclear GSTO1 localization (dark bars). ** and *a* indicate a significant difference from healthy (*P* < 0.001) or IBD (*P* < 0.01) subjects. In each group, bars represent the mean ± SE. **Panel B.** photos of lymphocytes counterstained with propidium iodide and visualized using filters for TRITC (1–4) or FITC (5–8). GSTO1 negative (not expressing the enzyme) lymphocyte (photo 5, left); lymphocytes expressing GSTO1 at nuclear level (photo 5, white arrow and photo 6); lymphocytes show cytoplasmic GSTO1 localization (photos 7 and 8). 1000 × magnification.

As shown in Figure 4A, healthy (22.18 ± 1.62%) and IBD subjects (16.75 ± 1.03%) display the lowest GSTO1 expression levels. Polyps (27.94 ± 4.20%) and CRC (34.35 ± 4.29%) patients, on the opposite, show the highest levels. Anyway, contrast analysis indicates that CRC category is significantly different from healthy subjects (P < 0.001) and that both polyps and CRC have higher levels of GSTO1 expression than IBD (*P* < 0.01). A similar trend was observed for the fraction of positive cells that express the enzyme at nuclear level. Mean values did not differ between healthy (38.91 ± 2.39%) and IBD subjects (28.39 ± 5.71%). Polyps (41.97 ± 5.84%) show significantly (*P* < 0.01) higher levels than IBD, indicating the direction of the nuclear relocation, which reaches the highest level in CRC samples (53.98 ± 6.83%, *P* < 0.01 vs. healthy and IBD subjects). Once again, sex and age did not affect GSTO1 expression and localization.

#### MN in mononucleated lymphocytes

**Figure 5:**
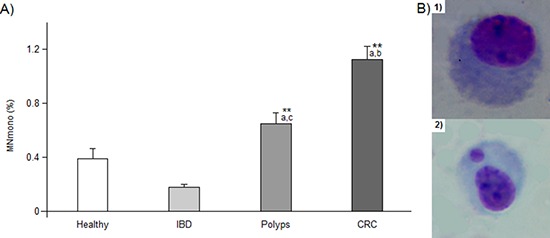
MNmono results **Panel A.** percentage of mononucleated lymphocytes bearing a micronucleus. ** or *a*, *b* and *c* indicate a significant difference (*P* < 0.001) from healthy subjects or from IBD, polyps and CRC patients, respectively. In each group, bars represent the mean ± SE **Panel B**: photos of lymphocytes (Giemsa staining), without micronucleus (1) and with a MN (2). 400x magnification.

Under forced cytodieresis inhibition condition, MN frequencies in mononucleated cells are 0.39 ± 0.08% and 0.18 ± 0.02% in healthy and IBD subjects, respectively, these values being not different from each other. In the polyp group, we observed a significant increase in the level of MN (0.65 ± 0.08%, *P* < 0.001) which doubles in the lymphocytes from CRC patients (1.12 ± 0.10%, *P* < 0.001 vs. healthy donors and IBD, *P* < 0.01 vs. polyps) (Figure 5A). Sex and age did not produce any effect on data variability.

## DISCUSSION

### Genomic damage markers

We used two markers to evaluate the genomic damage in subjects affected by pathologies leading to CRC: γH2AX as marker of DSB and the presence of micronuclei in mononucleated lymphocytes to assess predisposition to malsegregation events. Together, the markers give an overview of genomic instability in the progression to CRC including IBD as predisposing pathology, and polyp as pre-cancerous state. In general we observed a growing genomic stress, which is supported by the increase in the two markers. If in the traditional adenoma-carcinoma sequence the increase of genomic instability is evident, the relationship of IBD with this event seems to be more complex. The inflammatory state of IBD is not able to induce H2AX phosphorylation in tissue, suggesting that in this condition there is no threat to the maintenance of genomic stability. The low γH2AX level in IBD colorectal biopsies we observed seems to be not in line with the findings from Risques who reported increased γH2AX levels [20]. However, this is probably because our IBD samples, obtained from patients undergoing their first diagnosis, are indicative of a shorter period of inflammatory illness than the one known to cause an increase in CRC development risk [3]. When considering what happens in lymphocytes, in line with the active role that this cellular type plays in autoimmune diseases [21], we obtained the highest γH2AX levels. A persistent activation of the T lymphocytes can explain the γH2AX positivity, but can also indicate that lymphocytes undergo the effect of a systemic inflammatory state, known to generate ROS. The extent of γH2AX response in damaged lymphocytes in IBD is similar to the one observed in lymphocytes from healthy donors, indicating that in IBD the amount of cells which participates in the pathologies and shows the effect of the immune system activation is only numerically increased. In lymphocytes from our IBD patients, there is no indication of genomic instability connected to malsegregation events. Circulating blood cells can be therefore considered more sensitive in responding to a systemic genotoxic condition than the affected colon mucosa, and this is in line with a similar response found in IBD mouse models [22]. A concordance in γH2AX response between colon cells and lymphocytes is found in the pre-cancerous state and in CRC. In polyps, in fact, we found a 3-fold or at least 10-fold increase in γH2AX levels in tissues and lymphocytes, respectively. Interestingly, we also observed an increase in the extent of damage in the lymphocytes which reaches the amount of almost 4 DSB per positive cell. Furthermore, a significant increase in MNmono frequency indicates the existence of an association between the pathological state and alterations in chromosome segregation, probably due to the exposure to a hostile environment in polyp mucosa. The evolution of polyp to CRC seems to be, for several aspects, a prosecution of a growing genomic instability, especially in lymphocytes where the γH2AX levels increased in comparison with those found in polyps. An important difference is observed in the extent of internal lymphocyte damage, as this falls down to levels similar to those of healthy donors. This may indicate a decreased involvement of immune system in CRC, which becomes tolerant and unable to efficiently counteract the tumor growth. Growing neoplastic tissues, in fact, are known to escape the immune system control through several mechanisms such as the production of molecules inhibiting immune cellular activity, loss of expression of tumoral antigens or expression of FasL ligands which induce apoptosis in these cells [23], [24]. A low level of γH2AX foci in damaged lymphocytes suggests that they became unable to correctly sense the increasing genomic stress, probably as the consequence of an attenuated DDR activation. In colon tissue, we found a delay in γH2AX response with respect to lymphocytes, as the levels of this marker start increasing only from the pre-cancerous state and then stabilize in CRC. Interestingly, we also observed a great heterogeneity in γH2AX response within polyps and CRC, which is not present in the healthy and IBD groups. In particular, one fourth of CRC samples showed levels of phosphorylation 5-fold lower than in healthy tissue biopsies; on the opposite, the lower quartile delimiting value in polyps, is the highest among the studied categories. Basing on these data, a subgroup of subjects showed a decreased γH2AX response in neoplastic tissue, in connection to a DDR activity loss. The frequency of mononucleated lymphocytes bearing a MN doubled, passing from the pre-cancerous condition to CRC, confirming again the existence of an association between the neoplastic colon transformation and the impairment of chromosome segregation apparatus. According to our findings, increased MN levels were found in subjects affected by polyps and CRC [25]. In addition, it has been observed the existence of some diffusible factors in plasma from CRC patients, able to induce MN formation in lymphocytes of healthy donors [26].

#### GSTO1

Even though the precise role of GSTO1 has not been clarified yet, the enzyme's capacity of reducing dehydroascorbate, promoting glutathionylation against the irreversible oxidation of thiol protein groups as well as its expression in response to cellular stress, suggests a defensive role for this enzyme [15], [27]. Additionally, GSTO1 may be involved in regulation of several protein targets through the de/glutathionylation activity [17]. As previously reported [28], we found a high level of expression of GSTO1 in healthy colon tissue, especially at cytoplasmic level. In IBD tissues, GSTO1 expression decreases but there is a remarkable increase in its nuclear localization and function, and this can be connected to the absence of γH2AX positivity. In IBD lymphocytes, GSTO1 does not show substantial changes in its expression and nuclear localization. The lack of GSTO1 defensive function, combined with the effects of IBD condition, facilitates the accumulation of DNA insults which may evolve into DSB, thus explaining the high γH2AX levels found in lymphocytes. The GSTO1 loss of expression prosecutes in the pre-cancerous biopsies where we observed the halving of the positive area but a normal level of nuclear localization. In lymphocytes, GSTO1 continues to be expressed at levels similar to those from healthy donors, and also the fraction of cells with nuclear localization remains stable. The polyp formation may be direct or derive from a previous prolonged condition of IBD but, in both cases, it requires a relatively long time to complete. The gradual and prolonged decrease of GSTO1 expression, the lack of nuclear localization, in the context of an increasing cellular stress (due to the pathology itself and to the immune system activity), reflects in the high γH2AX positivity seen in the tissue. Neoplastic colon mucosa has a similar level of GSTO1 expression but a higher nuclear localization if compared to the pre-cancerous state. Overall, our findings are in agreement with the observed loss of expression of GSTTs in adenomas and tumor of the gastrointestinal tract [29], and with the increase in nuclear localization found in Barrett's oesophagus biopsies [18]. Furthermore, tumor cell lines overexpressing GSTO1 have been reported to resist to cis-platinum treatment through activation of the Akt and ERK 1/2 survival pathways [27]. In lymphocytes from CRC donors, GSTO1 expression is significantly increased and more than half of the cells express it at nuclear level. Taken together, these results indicate that colon cells are gradually losing GSTO1 defense activity in the pathological conditions, but this does not impair its nuclear function, which is otherwise promoted. From the pre-cancerous state, the decrease in GSTO1 expression is not sufficient to counteract the effects of the increasing cellular stress which lasts longer than in IBD, and this can explain the general increase in γH2AX seen in the biopsies. In peripheral blood lymphocytes, the levels of GSTO1 expression and localization remain stable until cancer formation, which presumably represents a stimulus for induction and nuclear relocation of the enzyme.

## CONCLUSIONS

In the present study, evaluation of genomic instability and cellular stress on colon biopsies and lymphocytes in colorectal pathologies delineates a growing level of DNA damage in the form of DSB and chromosome missegregation, accompanied by a loss of activity of an antioxidant defense due to change in GSTO1 expression and localization. The position of IBD in the sequence to CRC is particularly complex, as this pathology, in the first steps of its development, seems to constitute an alone standing condition. It is likely that the relatively short period of the pathology suffered by the patients here analyzed, does not represent a concrete risk for development of CRC. In IBD, in fact, the activity of antioxidant defense systems are still active in protecting DNA from ROS and RNS, which are physiologically produced in this pathology. This translates in a low level of DSB in cell tissue as well as within lymphocytes, without induction of malsegregation events. Anyway, the increase in damaged cells indicates an involvement of lymphocytes in sensing the effects of this pathology, which is in line with the idea of the induction of a systemic genotoxicity in the inflammatory state. This type of response, in particular H2AX phosphorylation, has been found also in the other pathologies analyzed as lymphocytes display high levels earlier than colon tissue. The condition of polyp and CRC are characterized by the increase of DSB and of chromosome malsegregation events in both colon and lymphocytes. In addition, as the level of DDR activation found in CRC biopsies is differentiated among patients, the γH2AX assay can help in recognizing which ones are probably going to positively respond to chemo/radiotherapy. The growing genotoxic stress is accompanied, and probably at least partially caused, by the loss of GSTO1 antioxidant defenses that, even if still working at nuclear level, may be quantitatively insufficient to counteract the effects of this condition. In particular, the weakness to cellular stress found in polyps and cancer tissues may be used in the development of therapies taking advantage of it to selectively hit cancer cells without affecting cell systems not losing this defense.

The evaluation of genomic damage and cellular stress associated to colorectal pathologies in peripheral circulating cells other than in colon tissue may be of clinical relevance.

## MATERIALS AND METHODS

### Study populations (colorectal biopsies and peripheral blood lymphocytes collection)

In collaboration with the U.O. Anatomia e Istologia 2, AOUP (Azienda Ospedaliera Universitaria Pisana), we retrospectively identified and included in the biopsy study 91 subjects who were subdivided according to the previously mentioned pathological classes, as indicated in Table 1. We studied 13 healthy tissue samples deriving from surgical resection margins, 20 IBD biopsies (11 ulcerative colitis and 9 Crohn's disease samples), 28 adenomas (including 8 hyperplastic polyps and 20 adenomas of various dysplasia grades) and 30 sporadic CRC cases, collecting data regarding age, sex, pathology subtype and anatomic provenience. From each donor, 4 paraffin-embedded sections from a colorectal biopsy were obtained and were subsequently analyzed for γH2AX and GSTO1 expression.

**Table 1:** Demographic composition and biopsy characteristics of tissue sample donors

Factor		Healthy	IBD	Polyps	CRC	Total
Sex*	M	6	12	15	16	49
	F	7	8	13	14	42
Age*^1^		78.7 ± 13.7	52.3 ± 15.5	66.1 ± 11.2	76.4 ± 11.5	68.3 ± 15.9
Biopsy type*^2^			11 UC	20 D		
			9 CD	8 H		
Biopsy provenience*^3^	P	6	1	10	8	25
	D	4	13	12	13	42
	R	3	6	6	9	24

* M and F indicate males and females, respectively.

*1 Age values, expressed in years, are the mean ± S.D. of each group.

*2 UC = ulcerative colitis; CD = Chron's disease; D = dysplasic polyps; H = hyperplastic polyps.

*3 Biopsy provenience indicates the anatomical district from which samples were obtained: proximal colon (P) (cecum, ascending colon, hepatic flexure, transverse colon), distal colon (D) (splenic flexure, descendign colon, sigma) and rectal colon (R).

Heparinised blood lymphocyte samples were obtained by venipuncture from 75 subjects, categorized as described above, who attended the U.O. Chirurgia ed Endoscopia (Ospedale Lotti Pontedera, Azienda USL 5 Pisa) or the U.O. Gastroenterologia (Ospedale Cisanello, AUOP). In particular, samples were obtained from 20 healthy donors, 15 IBD affected subjects, 20 polyps and CRC patients. The demographic composition of the population studied in this part of the work is indicated in Table 2. For each blood sample, γH2AX, GSTO1 and MNmono analysis were performed.

**Table 2:** Demographic characteristics of peripheral blood donors

Factor		Healthy	IBD	Polyps	CRC	Total
Sex*	M	8	6	9	10	33
	F	12	9	11	10	42
Age*^1^		50.8 ± 3.7	41.6 ± 4.5	66.6 ± 2.4	72.2 ± 2.9	58.3 ± 2.2

* M and F indicates males and females, respectively.

*1 Age values, expressed in years, are the mean ± S.D. of each group.

Both in tissue and blood sampling, subjects who underwent treatments known to interfere with the studied markers (e.g. anticancer drugs assumption or immuno-suppressive therapy) were excluded from the analyzed cohorts. An informed consent was obtained from each participant included in the study and all information was processed in anonymous way. The protocol was approved by the Azienda Ospedaliero-Universitaria Pisana ethical committee.

### Immunohistochemistry (IHC) in colorectal biopsies

Evaluation of γH2AX or GSTO1 was performed analyzing, separately for each marker, two sections per subject using a standardized immunohistochemistry protocol. Briefly, paraffin-embedded tissue sections were deparaffinized in pure xilene for 20 minutes, gradually hydrated in an ethanol series (100%, 95%, 70%) and then kept in distilled water. Antigen unmasking was performed in citrate buffer (10 mM, pH 6.0) keeping sections in water bath for 50 min at 98°C; this step was followed by 5 min of room temperature (RT) cooling. Endogenous peroxidases were inhibited incubating sections in a H_2_O_2_ solution (3% v/v) for 10 min at RT. Sections were rinsed in PBS 1X and incubated for 1 h at RT in a permeabilization solution (PS) consisting of inactivated fetal bovine serum (FBS, 10% Invitrogen, Milano, Italy), and Triton-X 100 (0.3%, Sigma-Aldrich, Milano, Italy) in PBS. Both primary antibodies (γH2AX: Abcam, Prodotti Gianni, Milano, Italy; GSTO1 was a generous gift of Dr. Simona Piaggi, Pisa University) were diluted in PS at 1:300. Sections were then covered with parafilm and incubated at 4°C overnight. Samples were rinsed in PBS and the horseradish peroxidase-labeled secondary antibody (Sigma-Aldrich, Milano, Italy) was applied at a working dilution of 1:100. Sections were then covered with parafilm and incubated for 1 h at RT. Samples were rinsed in PBS and stained with a diamminobenzidine-urea (DAB) solution following the manufacturer instructions (Sigma-Aldrich, Milano, Italy), for 12 min. After the staining reaction was arrested in distilled water, sections were counterstained with haematoxilyn and rinsed in distilled water. Finally, sections were dehydrated in histolene, mounted with DPX mounting medium and covered with cover slips.

**Figure 6:**
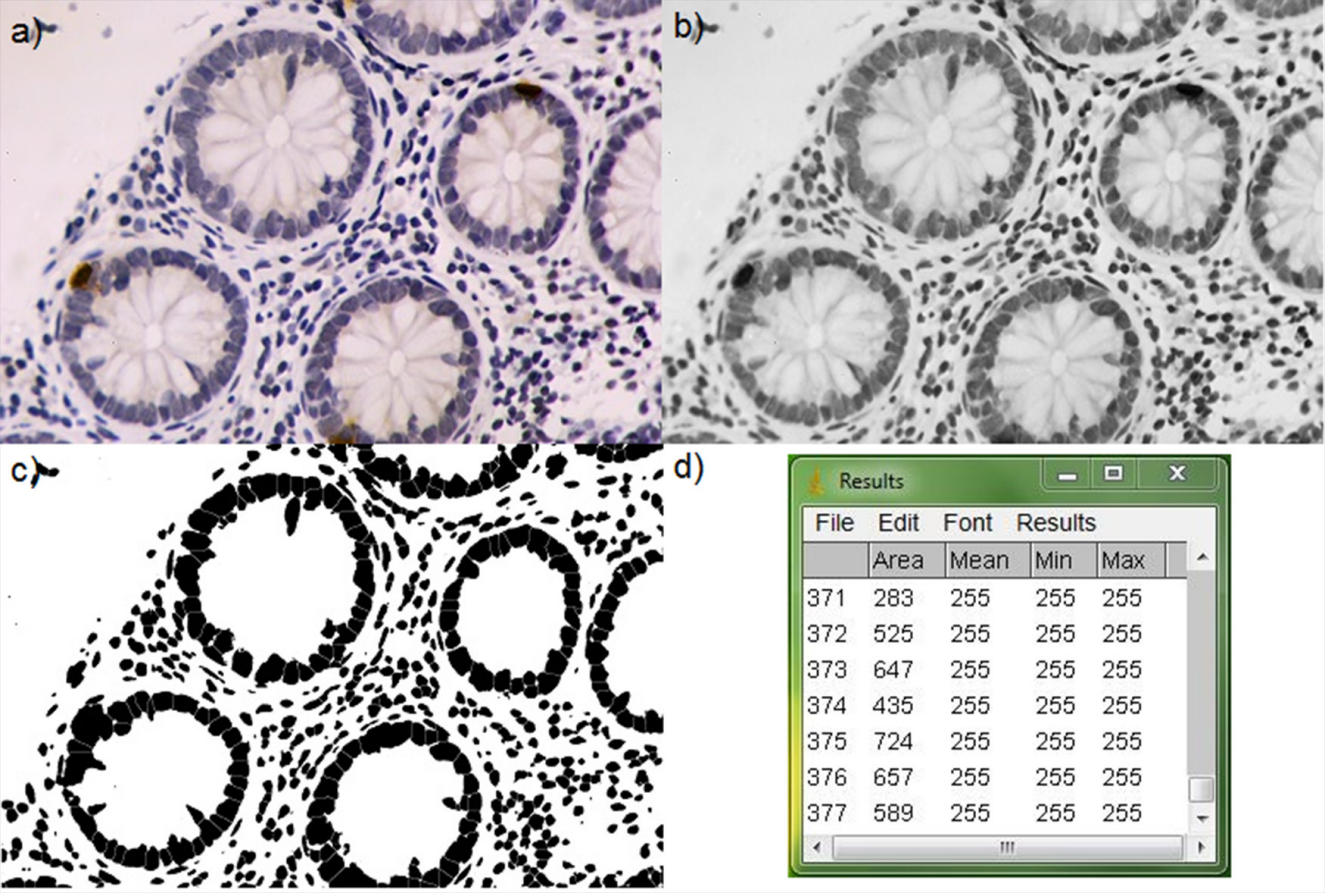
Image analysis for γH2AX quantification in tissue **A.** original image, **B.** gray-scale image, **C.** image after threshold setting and segmenting, and **D.** nuclei counting, after proper size definition. 200x magnification.

**Figure 7:**
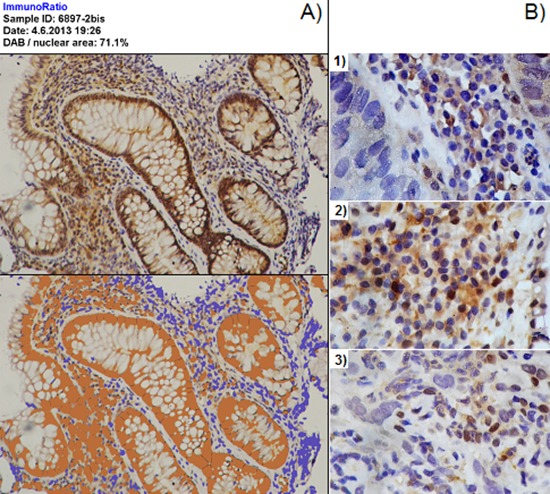
Image analysis for GSTO1 in tissue **Panel A.** use of ImmunoRatio for quantification of GSTO1 expression. The original image and the corresponding plug-in output, quantified as positive DAB area, are shown at the top and the bottom of the panel, respectively. 100x magnification. **Panel B.** quantification of GSTO1 localization through fields scoring; example of 1) cytoplasmic field (assigned score 0), 2) field with mixed localization (assigned score 0.5), and 3) field with prevalent nuclear localization (assigned score 1). 400x magnification.

For both γH2AX and GSTO1 evaluation, a quantitative analysis using a computerized image-based method was performed. Images were obtained using a Nikon optical microscope equipped with a reflex digital camera (D60, Nikon). In the case of γH2AX, we proceeded as follows. Two series of 20 pictures of not overlapping fields were obtained from each specimen, starting from opposite extremities of the section (40 pictures per section, 80 pictures per each subject). Pictures were randomly taken, using a 20x objective (magnification of 200x), and non–epithelial biopsy portions were excluded. Images were imported on computer and processed using the ImageJ software (download at http://imagej.nih.gov/ij/). Each RGB-color image was converted into 8-bit gray-scale image. Then an adequate threshold level was set up in order to transform the gray-scale image into a black and white image, where black stains correspond to the darkest gray stains (nuclei) in the preceding image. Stains were segmented using the specific program algorithm in order to separate partially overlapping nuclei. Once we coherently defined size range for stains, nuclei were counted by the program (Figure 6A–D). The number of positive nuclei (showing brown staining) was manually scored for each photo and the results were expressed as percentage of γH2AX positive cells. As for GSTO1 we observed a higher positivity level than for γH2AX, we proceeded using the ImmunoRatio plug-in of the ImageJ software. This plug-in is optimized to evaluate the percentage of positive area on images of sections stained with DAB and haematoxilyn [30]. We obtained 2 series of 10 pictures per section (20 images per section and 40 per subject) using the criteria already described with the exception of decreasing the magnification at 100x (objective 10x). After the proper calibration of the color thresholds required by the plug-in, the output is expressed as GSTO1 positive area (%) (Figure 7A). Localization of the enzyme in biopsies was quantified using 10 pictures obtained at 400x magnification (40x objective) per each section (20 pictures analyzed per each subject) giving them a score of 0 or 1 when enzyme localization was prevalently cytoplasmic or nuclear, respectively; in the case of mixed localization, 0.5 was added for the field (Figure 7B). The sum of scores assigned to each field was divided by the number of fields analyzed (10) and expressed as percentage of GSTO1 nuclear fields.

### Peripheral blood lymphocyte culturing and harvesting

Primary lymphocytes cultures were obtained from heparinised whole blood samples processed within 24 h from collection. At least six cultures (two for each analysis performed) were assessed from each subject. 0.3 ml of blood were added to 4.7 ml of RPMI-1640 culture medium (Invitrogen, Milano, Italy) supplemented with 20% FBS, 1% antibiotics and antimycotics and 1.5% phytohemagglutinin (Invitrogen, Milano, Italy). Cultures were kept at 37° for a variable time depending on the specific analysis requirements. For γH2AX analysis, cultures were immediately harvested in order to avoid lymphocytes stimulation, while for GSTO1 immunostaining they were harvested after 24 h proliferation. For assessment of MN induction in mononucleated cells, cultures where harvested after 72 h of incubation as described in the appropriate subsection (see below). In all the analyses performed, lymphocytes harvesting was performed using the same protocol described elsewhere [31]. Briefly, cells were initially precipitated by a 5 min centrifugation at 2100 rpm. After replacing the medium with 5 ml of hypotonic solution (KCl, 0.075 M), cells were pre-fixed in 0.4 ml of 5:3 acetic acid:methanol, re-centrifuged and the obtained pellet was resuspended in pure methanol for sample storage at −18°C.

### Immunofluorescence (IF) analysis of γH2AX and GSTO1

The evaluation of γH2AX and GSTO1 expression in lymphocytes was performed using the same IF protocol described elsewhere [31]. Briefly, methanol was removed, cells were fixed twice in methanol:acetic acid 3:1 or 8:1 for γH2AX or GSTO1 analysis, respectively, and the obtained pellet was dropped onto clean glass slides and left drying. Slides were then rinsed three times in PBS and incubated 1 h at RT in PS (see IHC procedure), then covered with primary antibody diluted in PS at 1:50 for anti-γH2AX (Cell Signaling, Euroclone, Milano, Italy) and 1:200 for anti-GSTO1. Slides were covered with parafilm and incubated overnight at 4°C. At this point, slides were rinsed in PBS three times and a DyLight488 labeled secondary antibody (Pierce, Euroclone, Milano, Italy) was applied at a working dilution of 1:200, then they were covered with parafilm and incubated at RT for 2 h. Slides were then rinsed in PBS and nuclei were counterstained with 0.4 μg/ml of propidium iodide in antifade solution; they were covered with cover slips and stored at 4°C until fluorescence observation. For each subject, four slides were analyzed on a Nikon-Optiphot-2 fluorescence microscope equipped with filters for FITC and TRITC using an 100x immersion-oil objective (magnification 1000x). Two slides were used for both γH2AX and GSTO1 analyses. For both markers, 400 cells were manually scored on each slide (800 cells per subject). In the case of γH2AX, we counted the overall number of positive cells (showing at least one fluorescent green-yellow spot) as well as the total amount of γH2AX nuclear foci in the considered cell population (Figure 3C). This allowed us to express γH2AX levels in blood samples as: 1) percentage of γH2AX positive cells, 2) average number of γH2AX foci per cell and 3) average number of γH2AX foci per positive cell. GSTO1 expression was investigated counting the number of cells expressing the enzyme (i.e. cells showing green fluorescence around the red nucleus, a yellow spotted nucleus with no fluorescence in the cytoplasm or both) on the total cell population scored, and reporting the GSTO1 positive cells percentage. The fraction of cells expressing nuclear GSTO1 localization was calculated as the ratio, in percent, between the number of cells showing a yellow spotted nucleus and the total GSTO1 positive cells. Cells showing cytoplasmic and nuclear fluorescence were considered as having nuclear localization (Figure 4B).

### Analysis of MN frequency in mononucleated cells

To analyze MN frequency in mononucleated cells, we modified the classical CBMN assay protocol adding Cyt B 6 μg/ml at 24 h instead of 44 h. In fact, in previous experiments conducted in our laboratory, we observed that when cytodieresis was inhibited adding Cyt B at 24 h, we obtained higher MNmono frequency than at any other time (data not shown). In this way, we were allowed to detect differences between subjects even in absence of treatments increasing the occurrence of this event. Cells were then harvested as described above, and slides were stained with Giemsa (5%), rinsed in distilled water and left drying. For each subject, 1000 mononucleated lymphocytes were counted per culture (2000 cells in total) using a Nikon optical microscope equipped with a 40x objective (magnification 400x). The results were then expressed as the average percentage of mononucleated cells carrying one or more micronuclei (MNmono %) (Figure 5B).

### Statistical analysis

To evaluate both the effect of the pathology on each marker and differences within or among the four categories, we used multivariate ANOVA followed by multiple comparison analysis with Bonferroni's correction. Factors able to introduce additional variability (sex, age, anatomical biopsy provenience and pathology sub-type) were also considered. When necessary, association between two variables has been evaluated using first order regression analysis. In each analysis performed, data were expressed, at group level, as mean ± S.E. Statistical analysis was performed using STATGRAPHICS Plus version 5.1 software package (Statistical Graphics Corporation, 2001, Rockville, USA).

## References

[1] American Cancer Society (2014). Cancer Facts and Figures 2014.

[2] Fearon ER, Vogelstein B (1990). A genetic model for colorectal tumorigenesis. Cell.

[3] Dyson JK, Rutter MD (2012). Colorectal cancer in inflammatory bowel disease: What is the real magnitude of the risk?. World J Gastroenterol.

[4] Rogakou EP, Pilch DR, Orr AH, Ivanova VS, Bonner WM (1998). DNA double-stranded breaks induce histone H2AX phosphorylation on serine 139. J Biol Chem.

[5] Sedelnikova OA, Pilch DR, Redon C, Bonner WM (2003). Histone H2AX in DNA damage and repair. Cancer Biol Ther.

[6] Sak A, Grehl S, Erichsen P, Engelhard M, Grannass A, Levegrün S, Pöttgen C, Groneberg M, Stuschke M (2007). gamma-H2AX foci formation in peripheral blood lymphocytes of tumor patients after local radiotherapy to different sites of the body: dependence on the dose-distribution, irradiated site and time from start of treatment. Int J Radiat Biol.

[7] Scarpato R, Verola C, Fabiani B, Bianchi V, Saggese G, Federico G (2011). Nuclear damage in peripheral lymphocytes of obese and overweight Italian children as evaluated by the gamma-H2AX focus assay and micronucleus test. FASEB J.

[8] Redon CE, Nakamura AJ, Martin OA, Parekh PR, Weyemi US, Bonner WM (2011). Recent developments in the use of γ-H2AX as a quantitative DNA double-strand break biomarker. Aging.

[9] Bartkova J, Horejsi Z, Koed K, Krämer A, Tort F, Zieger K, Guldberg P, Sehested M, Nesland JM, Lukas C, Ørntoft T, Lukas J, Bartek J (2005). DNA damage response as a candidate anti-cancer barrier in early human tumorigenesis. Nature.

[10] Kirsch-Volders M, Plas G, Elhajouji A, Lukamowicz M, Gonzalez L, Vande Loock K, Decordier I (2011). The *in vitro* MN assay in. origin and fate, biological significance, protocols, high throughput methodologies and toxicological relevance. Arch Toxicol.

[11] Guccini S, Lombardi S, Pisani A, Piaggi S, Scarpato R (2012). Effect of spindle poisons in peripheral human lymphocytes by the *in vitro* cytokinesis-block micronucleus assay. Mutagenesis.

[12] Elhajouji A, Cunha M, Kirsch-Volders M (1998). Spindle poisons can induce poliploidy by mitotic slippage and micronucleates in the cytokinesis block assay. Mutagenesis.

[13] Decordier I, Cundari E, Kirsch-Volders M (2008). Survival of aneuploid, micronucleated and/or polyploid cells: crosstalk between ploidy control and apoptosis. Mutat Res.

[14] Gupta RK, Patel AK, Shah N, Chaudhary AK, Jha UK, Yadav UC, Gupta PK, Pakuwal U (2014). Oxidative stress and antioxidants in disease and cancer: a review. Asian Pac J Cancer Prev.

[15] Board PG, Coggan M, Chelvanayagam G, Easteal S, Jermiin LS, Schulte GK, Danley DE, Hoth LR, Griffor MC, Kamath AV, Rosner MH, Chrunyk BA, Perregaux DE (2000). Identification, characterization, and crystal structure of the Omega class glutathione transferases. J Biol Chem.

[16] Laborde E (2010). Glutathione transferases as mediators of signaling pathways involved in cell proliferation and cell death. Cell Death Differ.

[17] Menon D, Board PG (2013). A role for glutathione transferase Omega 1 (GSTO1–1) in the glutathionylation cycle. J Biol Chem.

[18] Piaggi S, Marchi S, Ciancia E, Debortoli N, Lazzarotti A, Saviozzi M, Raggi C, Fierabracci V, Visvikis A, Bisgaard HC, Casini AF, Paolicchi A (2009). Nuclear translocation of glutathione transferase omega is a progression marker in Barrett's esophagus. Oncol Rep.

[19] Kodym R, Calkins P, Story M (1999). The cloning and characterization of a new stress response protein. A mammalian member of a family of theta class glutathione s-transferase-like proteins. J Biol Chem.

[20] Risques RA, Lai LA, Brentnall TA, Li L, Feng Z, Gallaher J, Mandelson MT, Potter JD, Bronner MP, Rabinovitch PS (2008). Ulcerative colitis is a disease of accelerated colon aging: evidence from telomere attrition and DNA damage. Gastroenterology.

[21] Wallace KL, Zheng LB, Kanazawa Y, Shih DQ (2014). Immunopathology of inflammatory bowel disease. World J Gastroenterol.

[22] Westbroock AM, Wei B, Schiestl RH (2009). Intestinal mucosal inflammation leads to systemic genotoxicity in mice. Cancer Res.

[23] Spurrell EL, Lockley M (2014). Adaptive immunity in cancer immunology and therapeutics. Ecancermedicalscience.

[24] O'Connell J, Bennett MW, Nally K, Houston A, O'Sullivan GC, Shanahan F (2000). Altered mechanisms of apoptosis in colon cancer: Fas resistance and counterattack in the tumor-immune conflict. Ann N Y Acad Sci.

[25] Karaman A, Binici DN, Kabalar ME, Calikuşu Z (2008). Micronucleus analysis in patients with colorectal adenocarcinoma and colorectal polyps. World J Gastroenterol.

[26] Maffei F, Angeloni C, Malaguti M, Moraga JM, Pasqui F, Poli C, Colecchia A, Festi D, Hrelia P, Hrelia S (2011). Plasma antioxidant enzymes and clastogenic factors as possible biomarkers of colorectal cancer risk. Mutat Res.

[27] Piaggi S, Raggi C, Corti A, Pitzalis E, Mascherpa MC, Saviozzi M, Pompella A, Casini AF (2010). Glutathione transferase omega 1–1 (GSTO1–1) plays an anti-apoptotic role in cell resistance to cisplatin toxicity. Carcinogenesis.

[28] Yin ZL, Dahlstrom JE, Le Couteur DG, Board PG (2001). Immunohistochemistry of omega class glutathione S-transferase in human tissues. J Histochem Cytochem.

[29] de Bruin WC, Wagenmans MJ, Board PG (1999). Expression of glutathione S-transferase theta class isoenzymes in human colorectal and gastric cancers. Carcinogenesis.

[30] Tuominen VJ, Ruotoistenmäki S, Viitanen A, Jumppanen M, Isola J (2010). ImmunoRatio: a publicly available web application for quantitative image analysis of estrogen receptor (ER), progesterone receptor (PR), and Ki-67. Breast Cancer Res.

[31] Scarpato R, Castagna S, Aliotta R, Azzarà A, Ghetti F, Filomeni E, Giovannini C, Pirillo C, Testi S, Lombardi S, Tomei A (2013). Kinetics of nuclear phosphorylation (γ-H2AX) in human lymphocytes treated *in vitro* with UVB, bleomycin and mitomycin C. Mutagenesis.

